# Crystallization, Structure and Significantly Improved Mechanical Properties of PLA/PPC Blends Compatibilized with PLA-PPC Copolymers Produced by Reactions Initiated with TBT or TDI

**DOI:** 10.3390/polym13193245

**Published:** 2021-09-24

**Authors:** Lixin Song, Yongchao Li, Xiangyu Meng, Ting Wang, Ying Shi, Yuanxia Wang, Shengnan Shi, Li-Zhi Liu

**Affiliations:** 1 Advanced Manufacturing Institute of Polymer Industry, Shenyang University of Chemical Technology, Shenyang 110142, China; lxsong@syuct.edu.cn (L.S.); liyongchao970214@163.com (Y.L.); mengxiangyu170901@163.com (X.M.); wangting960207@163.com (T.W.); shiying@syuct.edu.cn (Y.S.); wangyuanxia@aliyun.com (Y.W.); 2 Shenyang Advanced Coating Material Industry Technology Research Institute Co., Ltd., Shenyang 110326, China; 18842350185@163.com; 3 College of Materials Science and Engineering, Shenyang University of Chemical Technology, Shenyang 110142, China

**Keywords:** polylactic acid/polypropylene carbonate blends, toluene diisocyanate, tetrabutyl titanate, crystallization property, thermodynamic properties

## Abstract

Poly (lactic acid) (PLA)-Poly (propylene carbonate) (PPC) block copolymer compatibilizers are produced in incompatible 70wt%PLA/PPC blend by initiating transesterification with addition of 1% of tetra butyl titanate (TBT) or by chain extension with addition of 2% of 2,4-toluene diisocyanate (TDI). The above blends can have much better mechanical properties than the blend without TBT and TDI. The elongation at break is dramatically larger (114% with 2% of TDI and 60% with 1% of TBT) than the blend without TDI and TBT, with a slightly lower mechanical strength. A small fraction of the copolymer is likely formed in the PLA/PPC blend with addition of TBT, and a significant amount of the copolymer can be made with addition of TDI. The copolymer produced with TDI has PPC as a major content (~70 wt%) and forms a miscible interphase with its own Tg. The crystallinity of the blend with TDI is significantly lower than the blend without TDI, as the PLA blocks of the copolymer in the interphase is hardly to crystallize. The average molecular weight increases significantly with addition of TDI, likely compensating the lower mechanical strength due to lower crystallinity. Material degradation can occur with addition of TBT, but it is very limited with 1% of TBT. However, compared with the blends without TBT, the PLA crystallinity of the blend with 1%TBT increases sharply during the cooling process, which likely compensates the loss of mechanical strength due to the slightly material degradation. The added TDI does not have any significant impact on PLA lamellar packing, but the addition of TBT can make PLA lamellar packing much less ordered, presumably resulted from much smaller PPC domains formed in the blend due to better compatibility.

## 1. Introduction

Polylactic acid (PLA) is a non-toxic, biodegradable and renewable semi-crystalline polymer, which is widely used in biomedicine, textiles, packaging and other fields [1,2,3,4,5,6,7,8]. However, PLA is rigid and lacks flexibility under room temperature due to its relatively high glass transition temperature (~60 °C), which limits its applications [8,9,10]. PLA is often modified by being blended with other polymers [11,12,13,14,15], as well as inorganic fillers [16,17,18], in order to enhance its crystalline ability and improve its mechanical and thermal properties. However, compatibilizers are required to improve the compatibility of PLA blend with other polymers [19,20,21]. Zhang et al. [20] used polyurethane elastomer prepolymer (PUEP) as the active compatibilizer to obtain super-toughened PLA/thermoplastic polyurethane (TPU) blends by reacting the isocyanate (*NCO*) groups in PUEP with the -OH groups on both sides of the PLA molecule.

Poly (propylene carbonate) (PPC) can be made by the copolymerization of propylene oxide and “greenhouse gas” CO_2_ as the raw materials, which alleviates the greenhouse effect and white pollution [22,23]. PPC is a relatively soft material with high elongation at break and can be added to PLA to improve the flexibility and thermodynamic properties of PLA [24]. The work carried out by Zhao [24] et al. shows that the elongation at break of PLA/PPC blends is much higher than neat PLA (1.9%). PPC and PLA are incompatible [25,26,27], which negatively impact its mechanical property. Phase separation in PLA/PPC blends was observed [25,26] and the modulus and tensile strength of the blend was reported lower than neat PLA. Flodberg [27] et al. studied the PLA/PPC blend with more than 30wt% of PPC, and noticed that severe phase separation could occur, resulting in a very low elongation at break and toughness. Therefore, it is very important to compatibilize the two components in the blend to improve the mechanical property. The blend can be compatibilized by adding a compatibilizer, such as, PLA-PPC block copolymer, changing the molecular structure of PLA and/or PPC, such as, grafting the polymer with different component [28,29], or adding additives to produce PLA-PPC block copolymer during the material processing. It is obvious that if the blend can be compatibilized well with addition of an additive, it would be relatively easy and cost effective, especially for industry applications. 

In fact, the compatibility of PLA and PPC can be improved by addition of additives [30,31]. Yao [30] et al. added MA as compatibilizer to PLA/PPC blend and found that MA can effectively enhance the compatibility between PLA and PPC. Qin [31] et al. used DCP as cross-linking initiator to initiate the reaction between PLA and PPC, which improved their compatibility and increased the elongation at break of the PLA/PPC blend by 200%. Polycarbonate/polyester blends undergo spontaneous transesterification during the melt-blending process [32]. However, the rate of transesterification is relatively low under general processing conditions. The addition of tetra butyl titanate (TBT) as the transesterification catalyst increases the reaction rate [33,34] and enhances the compatibility. The corresponding group exists at the end of the chain in PLA and PPC molecules. Thus, 2,4-toluene diisocyanate (TDI) can be used as a chain extender to connect the two molecular chains and improve their compatibility.

In this paper, the compatibilizing effects of TBT and TDI are studied, including the effects on crystallization dynamics, thermal and mechanical properties. As mentioned above, the PLA/PPC blend with more than 30wt% of PPC can have severe phase separation and lead to very low toughness and elongation at break. In this case, 70%PLA/PPC blend is selected for the study in the present work, and the main learnings can be leveraged to the blend with less PPC content.

## 2. Experiment

### 2.1. Materials

The poly (lactic acid) (PLA) (trade name 4032D) used in this study was obtained from NatureWorks (NatureWorks Inc., Blair, NE, USA). Poly (propylene carbonate) (PPC) (trade name 4701) was kindly supplied by the Changchun Institute of Applied Chemistry, Chinese Academy of Sciences (Changchun, Jilin, China). The chain extender 2,4-toluene diisocyanate (TDI) (chemically pure grade) was purchased from the Tianjin Hengxing Chemical Reagent Manufacturing Co., Ltd. (Tianjin, China). Maleic anhydride (MA) (chemically pure grade) was purchased from Beijing Chemical Industry Group Co., Ltd. (Beijing, China). Tetra butyl titanate (TBT) (chemically pure grade) was purchased from the Tianjin Guangfu Fine Chemical Research Institute (Tianjin China).

### 2.2. Sample Preparation

PPC has poor thermal stability and exhibits extreme thermal degradation above 150 °C. Therefore, the PPC was end-capped prior to the melt-blending process in order to obtain maleic anhydride-terminated polypropylene carbonate (PPC-MA) and thus improve its thermal stability [35,36]. 

The starting materials, PLA, PPC, TDI and TBT, were reactively blended in a RM-200ATorque Rheometer (Harbin Hapro Electrical Technology Co., Ltd., Harbin, Heilongjiang, China) under 180 °C at a rotor speed of 45 rpm for 7 min to prepare a series of PLA/PPC/TDI and PLA/PPC/TBT blends (Sample name and component contents are shown in Table 1).

### 2.3. Thermal Analysis

The melting and crystallization behavior of the blends were characterized using the TA Q200 Differential scanning calorimetry (DSC) (TA Instruments, USA) under a nitrogen atmosphere. About 5 mg of the composite material sample was weighed out into a crucible. The temperature was raised from 0 °C to 200 °C at a rate of 10 °C/min and kept constant for 5 min to eliminate thermal history, which was further lowered to 20 °C at the same rate. Finally, the temperature was raised to 200 °C at a rate of 10 °C/min, and the DSC data was collected for the study of the melting and cooling behavior of the blend. Heat flow was converted into heat capacity to determine the change of heat capacity at glass transition [37,38]. The crystallinity of the blend was calculated using Equation (1):

X_c_ = ΔH_m_/ΔH_0_ ×100% (1)

where X_c_ is the relative crystallinity of the sample, ΔH_m_ is the melting enthalpy of the sample, ΔH_0_ is the theoretical heat of fusion for a completely crystallized PLA with a value of 93.6 J/g [39].

### 2.4. Synchrotron Small-Angle X-ray Scattering (SAXS) Measurements

The weighed pellets were evenly placed in a mold. This polymer was melt-pressed at a pressure of 5 MPa under 200 °C for 5 min to form a sheet of 1 mm thickness. The sheets were subsequently cooled at room temperature without releasing the pressure. All the specimens were cut into rectangular strips with the dimensions of 0.1 × 1 × 1 cm^3^.

The synchrotron small-angle X-ray diffraction experiment was performed using synchrotron radiation with the λ value of 0.154 nm in Beamline 1W2A from the Beijing Synchrotron Radiation Facility (Beijing, China). The Mar 165-CCD detector with 2048 × 2048 pixels, 79.6 µm pixel size and (0.08 × 2048/2)^2^π area was used at a sample-detector distance of 3000 mm in the direction of the beam for the collection of SAXS data. The experimental samples were pasted on copper sheet clamping grooves with an insulating polyimide film. The temperature was raised from 0 °C to 200 °C at a rate of 10 °C/min and then kept constant for 10 min. Later, the temperature was lowered to 40 °C at a rate of 4 °C/min. The data was collected during the temperature variation process, and the background correction was performed before further analysis. The position of the peak, q_max_, is related to the long period L by Equation (2):

L = 2π/q_max_ (2)

where q is the scattering vector defined as q = 4π (sin θ)/λ, λ is the X-ray wavelength, and θ is the half of the scattering angle (2θ) [40].

### 2.5. Melt Index Testing

The MI was determined by a melt flow rate instrument (XNR-400, Chengde Jinjian Testing Instrument Co., Ltd., Chengde, Hebei, China) at 190 °C with a load of 2.16 kg. Each section was cut for 20 s. The sample strip was cooled, weighed and the average value was used to calculate the melt index by Equation (3):

MI = 600m/t(3)

where m is the mass of the material section (g) and t is the time (20 s).

### 2.6. Tensile Testing

The blends were made into tensile specimen by micro injection molding machine (WZS05, Shanghai xinshuo precision machinery Co., Ltd., Shanghai, China) according to the GB/T 1040–1BA standard. A universal testing machine (Instron 3365, USA) was used to test the tensile properties. Each sample was clamped at both ends and then stretched at a crosshead speed of 25 mm/min. The initial length l_0_ is 25mm. At least five specimens were tested for each sample to obtain an average for all the mechanical properties. All the mechanical properties were performed at room temperature.

## 3. Results and Discussion

**Figure 1:**
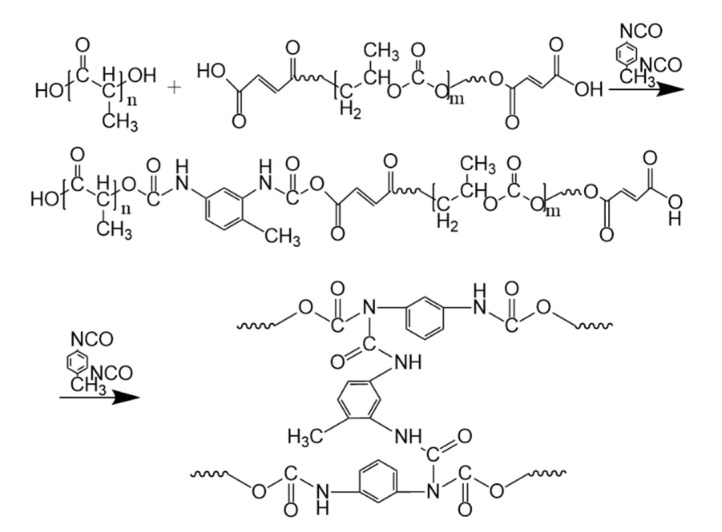
Chain extension reaction of PLA and PPC.

The addition of TDI and TBT to PLA/PPC blend can initiate the chemical reaction between PLA and PPC. The addition of appropriate amounts of TDI triggers the chain extension reaction between PLA and PPC (as shown in Figure 1). The hydroxyl groups in both PPC and PLA are dehydrogenated and polycondensed with the cyanate group of TDI. Carbamate groups are introduced due to chain extension, resulting in the incorporation of more flexible molecular groups of PPC into PLA [41].

**Figure 2:**
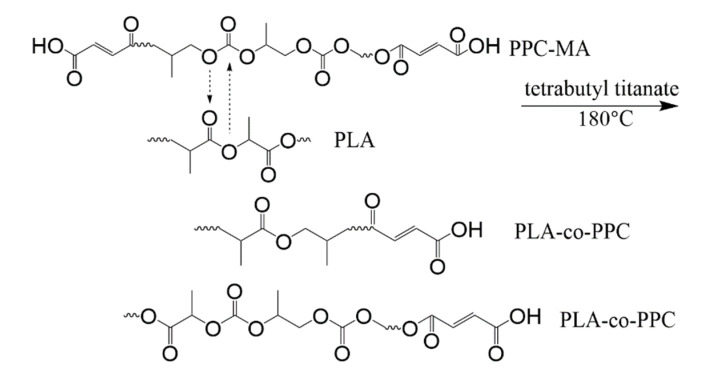
Transesterification reaction of PLA and PPC.

The reaction initiated with the addition of TBT is shown in Figure 2. Due to transesterification, the macromolecule breaks heavily, and the short fragment of PLA and PPC recombine randomly to produce PLA-co-PPC; thus, improving the compatibility between them. With more TBT added to the blend, the transesterification effect is more pronounced [42].

In the present work, melt index is characterized first for these blends (Table 2) to have a good understanding of the reacted materials, to see if there is any material degradation. It can be seen from Table 2 that the melt index of the PLA/PPC blend with addition of 1 wt% of TBT increases obviously, compared with the blend without TBT. With further increase in TBT content to 2%, the melt index increases significantly. The melt index of the blend with 3% of TBT increases to about 300 (vs. 15.6 for the blend without TBT), indicating severe degradation occurs in this sample. That is, when the TBT content is about 1% in the blend, the chain growth initiated by the transesterification and chain breaks up due to the degradation can be balanced to keep a relatively stable molecular weight. With 2% of TBT in the blend, the degradation outpaces the molecular weight increase by the transesterification, and appreciable decrease in molecular weight is noticed. With 3% of TBT in the blend, the degradation far outpaces the transesterification, leading to a sharply increased melt index. However, no degradation is observed for the blend with addition of TDI (not reported in literature neither), in fact, the melt index of the blend with 2% of TDI decreases sharply from 20.5 for the PLA/PPC blend to 4.5 with additional of 2% TDI, indicating significant increase in molecular weight due to the chain extension.

### 3.1. The Compatibility of PLA/PPC Blend and the Effects of Added TBT or TDI on the Compatibility

**Figure 3:**
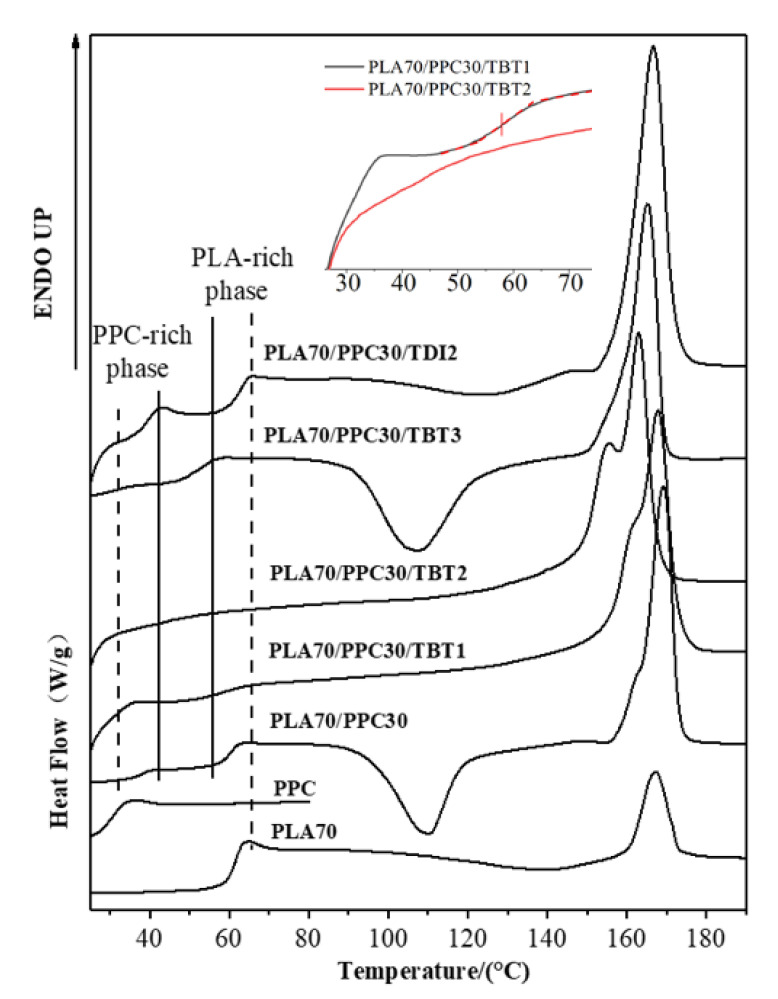
DSC heating thermograms of the studied samples at a rate of 10 °C/min (the samples were first heated to 200 °C and held for 5 min, and then cooled down to room temperature at a rate of 10 °C/min).

The glass transition of the neat PLA, the PPC and the PLA/PPC blends with TBT or TDI, were studied with DSC. The samples had been initially heated to 200 °C, held for 5 min, then cooled down to room temperature at 10 °C/min before the samples were heated up at 10 °C/min for measurement. The thermograms obtained during the 2nd heating process are shown in Figure 3 and the glass transition temperature for each sample is listed in Table 3. As seen from this figure that the glass transition temperature of the PPC and PLA is 30.5 °C and 61 °C, respectively. The PLA70/PPC30 blend shows two distinct glass transition temperatures at 37.5 °C and 59.8 °C, respectively, corresponding to the glass transition temperature of PPC rich phase and PLA rich phase, respectively. It is also noticed that the glass transition temperature of PLA rich phase in PLA70/PPC30 blend (59.8 °C) is slightly lower than the neat PLA (61 °C), while the glass transition temperature of PPC rich phase (37.5 °C) is appreciably higher than neat PPC (30.5 °C). The above results obviously indicates that the two polymers are not miscible, but the changes in Tg also suggest that the fraction of PLA in PPC rich phase is appreciably larger than the fraction of PPC in PLA rich phase. 

**Figure 4:**
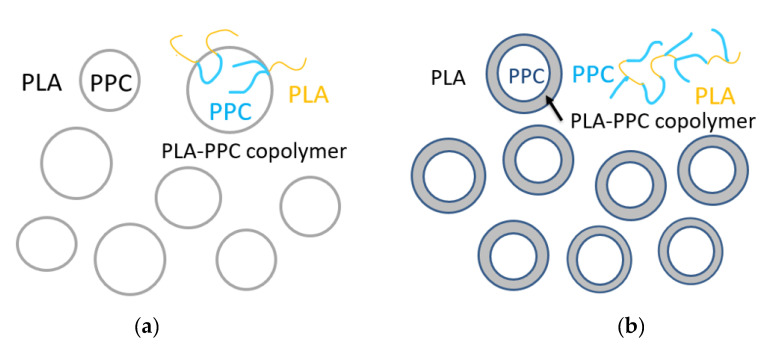
Schematic drawing of a small fraction of PLA-PPC block copolymer, obtained by the transesterification initiated with added TBT, act as the compatibilizer at the interface between PLA and PPC phases in PLA70/PPC30 blend with 1–2% of TBT (**a**), and the interphase between PLA and PPC phases, made of significant amount of PLA-PPC copolymer in PLA70/PPC30 blend with 2% of TDI due to the reaction of chain extension initiated by TDI (**b**).

It can be seen from Figure 3 and Table 3 that for the blend with 1% of TBT, the glass transition temperature of PPC rich phase (33.6 °C) is even lower than that of the PLA/PPC blend (37.5 °C). In fact, the glass transition temperature PPC rich phase in the blend with 2% of TBT is even lower (32.7 °C) than the blend with 1% of TBT. The fact that the Tg corresponding to PPC and PLA phases is still present indicates that the blends with 1–2% of TBT are incompatible with macrophase separation. Therefore, a small fraction of PLA -PPC block copolymer is likely formed in the PLA/PPC blend due to the transesterification with added 1–2% of TBT, and the block copolymer can act as the compatibilizer at the interface between PLA and PPC phases, as illustrated in Figure 4a. As the added TBT can lead to both the transesterification and degradation as discussed in previous section. The lower Tg for the PPC rich phase in the blends with 1–2% of TBT can be mainly attributed to the lower molecular weight of PPC due to the degradation effect, as seen in Table 2 for the increase in melt index. The Tg of the PLA rich phase in the blend with 1% of TBT is 57.9 °C, which is about 2 °C lower than that the PLA rich phase in the PLA/PPC blend, suggesting that the degradation to PLA is relatively limited. With 2% TBT added to the blend, the Tg of PLA phase is not well defined, as seen from the inset figure of Figure 3, but with 3% of TBT, a miscible blend is achieved as only one glass transition is observed at 51.8 °C. The significant degradation of the blend with 3% of TBT, especially the PPC component, can be one of the main reasons of why this blend becomes miscible. In fact, according to Fox Equation [43], the calculated single Tg of miscible mixture of 70%PLA/30%PPC is about 52 °C, consistent with what is observed in this work. 

The effect of added TDI on the PLA/PPC blend is quite different. As can be seen from the Figure 3 and Table 3 that three glass transitions are observed for this blend. Compared with the PLA/PPC blend, the changes in Tg of PPC rich phase and PLA rich phase in the blend with 2% of TDI are very different the blend with TBT. Firstly, the Tg of PPC rich phase in the blend with TDI (31.2 °C) is significantly lower than the PPC rich phase in the PLA/PPC blend (37.5 °C), indicating that the PLA fraction molecularly dissolved in the PPC rich phase in the blend with TDI is significant less than the PPC rich phase in the PLA/PPC blend. It is noted that based on the melt index of the reacted blends (Table 2), there is no degradation for the blend with TDI. Thus, the change in Tg of PPC rich phase can only be attributed to small amount of PLA molecularly dissolved in the PPC phase. Secondly, the glass transition temperature for the PLA rich phase in the blend with TDI is slightly higher than the PLA phase in the PLA/PPC blend, which is also different from the blends with TBT. Most importantly, another glass transition is observed at 39.4 °C, which presumably represents a miscible phase of PLA-PPC block copolymer. In fact, block copolymers typically have microphase separation and distinct Tg corresponding to each phase can be detected. Only one glass transition observed for the copolymer can occur when the incompatibility degree of the copolymer is low and microphase separation is absent. With Fox equation, the estimated PLA to PPC ratio for this copolymer is about 30:70. The PLA-PPC block copolymer formed in PLA/PPC blend with 2% of TDI is likely to have complicated grafting structure as shown in Figure 4b, which leads to a single miscible phase consisting of the copolymer with its own Tg. The fact that a distinct Tg observed for this block copolymer also indicates that a significant amount of PLA-PPC copolymer in PLA70/PPC30 blend with 2% of TDI is formed due to the reaction of chain extension initiated by TDI. As a result, the third phase is formed in the blend, that is the interphase between PLA and PPC phases, as shown in Figure 4, which is supposed to be very beneficial to the mechanical properties of this blend. The fact that the glass transition temperature of the PLA phase in the blend with TDI is even higher than the neat PLA and the Tg of PPC phase in the blend almost remains the same as the neat PPC could imply that the copolymerization of PLA and PPC with added TDI catalyst preferably occur among the fractions of PLA and PPC which have a relatively lower molecular weight.

On the other hand, it can be seen from Table 3 that for the PLA70/PPC30 blend, the heat capacity for the PPC-rich phase is only one third of the neat PPC homopolymer, which is consistent with one third of PPC in the blend and suggests that PLA molecules in the PPC rich phase contributes little to the heat capacity at the glass transition temperature of PPC rich phase. However, the heat capacity for the PLA-rich phase changes very little compared with the neat PLA homopolymer (0.61 vs. 0.59) even though the PLA content is only 70% in the blend, meaning that the heat capacity of the PLA-rich phase is significantly larger than the neat PLA. The significantly larger heat capacity of the PLA rich phase likely associates with a significantly larger PLA segment motion at its glass transition temperature due to the inclusion of soft PPC molecules in the PLA rich phase. It can also be seen from Table 3 that for the incompatible blends with 1–2% of TBT, the heat capacity at glass transition for the PPC rich phase increases more than 20% compared with the blend without TBT, which can be attributed to significantly increased mobility of PPC segments due to PPC degradation. In fact, the heat capacity at glass transition for the PPC rich phase in the blend with 2% of TDI shows little change compared with the blend without TDI or TBT (Table 3), as no PPC degradation occurs when TDI is added into the blend. The blend with 3% of TBT is miscible and the heat capacity for the single glass transition is about the average heat capacity of PLA and PPC, evaluated based on the blend composition (Table 3). As also listed in Table 3, the heat capacity of PLA rich phase for the blends compatibilized with TBT or TDI is significantly smaller than the blend without TBT and TDI; this can be interpreted as follows. Much smaller and significantly more PPC domains are likely dispersed in the PLA matrix for the compatibilized blends, which may lead to more conformational constraints for PLA molecules, so that the mobility PLA segments is decreased at the glass transition of PLA rich phase. The heat capacity of the PLA-PPC miscible copolymer phase is 0.36 (J/g·°C, Table 3), which is also about the averaged heat capacity based on the estimated copolymer composition (30% PLA/PPC).

### 3.2. Effects of Added TBT and TDI on the Non-Isothermal Crystallization Dynamics and Melting Behavior of PLA/PPC Blend

**Figure 5:**
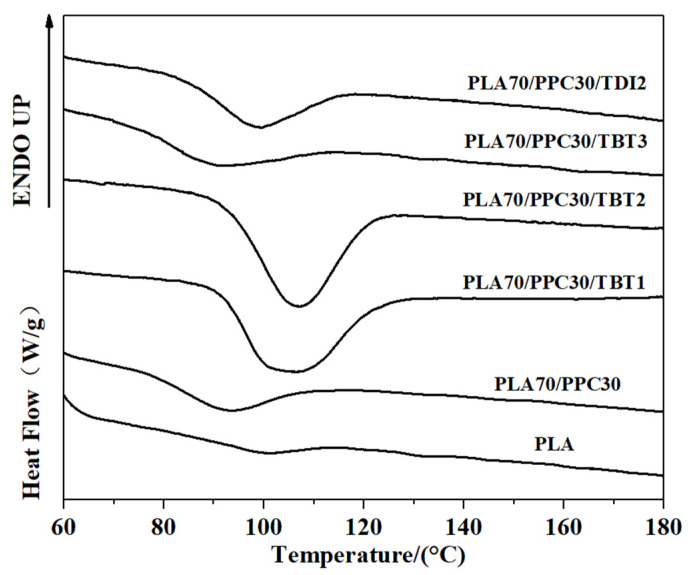
DSC cooling curves of the PLA/PPC blends.

The non-isothermal crystallization of PLA and their blends were studied with DSC during a cooling process. The cooling traces of PLA, PPC and their blends are shown in Figure 5; the crystallization temperature (T_c_) and cooling enthalpy (H_c_), as well as the crystallinity of PLA component, are listed in Table 4. The reported enthalpy value for PLA with 100% crystallinity is 93.6 J/g [39], which is used for the evaluation of PLA crystallinity in the present work. A faint crystallization peak of neat PLA with enthalpy of 0.83 J/g is observed during the cooling at a rate of 10 °C/min (corresponding a crystallinity of 0.92%), indicating that the crystalline ability of neat PLA is very weak during the cooling process. However, the blend with 30 wt% PPC (PLA70/PPC30) shows a significantly larger crystalline peak during the cooling (Figure 5) with ΔH_c_ = 2.74 J/g, which corresponds a PLA crystallinity of 4.17%. The enhanced crystalline ability of PLA in this blend can be attributed to the partial compatibility between the PLA and PPC. The lower glass transition temperature of PPC (30.5 °C) than PLA (61 °C) can lead to an increased mobility of PLA component in the blend which, in turn, leads to improved crystalline ability of PLA component. In fact, the compatibility of the blend is very limited, but a small fraction of PPC in PLA region can increase the mobility of the PLA to enhance the crystalline ability of PLA during the cooling [42]. The significantly lower crystallization temperature of the PLA/PPC blend with 30 wt% of PPC than the neat PLA (in Figure 5), is likely due to the slower crystallization of PLA in the blend, hindered by the small fraction of PPC in PLA rich phase [44]. 

It should be noted that the crystallization peak of the PLA70/PPC30/TBT1 blend with addition of 1 wt% of TBT shows a much stronger crystalline ability during the cooling process than the blend PLA70/PPC30 without TBT, as shown in Figure 5. As listed in Table 4 that the crystallization temperature of the PLA70/PPC30 is at 92.9 °C, while it increases to 106.5 °C with addition of 1% of TBT. On the other hand, the crystallinity of PLA component in its blend with 30% of PPC is only 4.17%, while it increases sharply to 42.31% with added 1 wt% of TBT (Table 4). With further increase in TBT to 2 wt%, the crystallization temperature remains unchanged and PLA crystallinity increases slightly as seen from Table 4. However, when TBT content increases to 3 wt%, the crystalline ability is dramatically weaken as shown in Figure 5 with a significantly lowered crystallization temperature (9 °C lower than the neat PLA), and much lower PLA crystallinity. That is, the blend with 3 wt% of TBT behaves very similar to the PLA70/PPC30 blend without TBT in terms the crystallization during the cooling process. This can be attributed to the formation of excessive amount of the PLA-PPC block copolymer in the blend with 3 wt% of TBT. The excessive amount of the copolymer can make the PLA rich-phase much smaller and hence weaken the crystalline ability of PLA component. Figure 5 also shows that crystallization dynamics and the crystallinity of the blend with 2 wt% of TDI is very similar to the blend with 3 wt% of TBT.

The subsequent heating the PLA, PPC and the blends are also investigated in the present work. The DSC melting thermograms are shown in Figure 3; the cold crystallization temperature (T_cc_) of PLA component, its corresponding change in enthalpy (H_cc_), the melting enthalpy of (H_m_) of PLA crystals, as well as the crystallinity of PLA component are all listed in Table 5. As shown in Figure 3, the neat PLA exhibited a broad cold crystallization with a peak temperature near 140 °C as the crystallization during the cooling process is very limited. Compared with the neat PLA homopolymer, the cold crystallization peak of the PLA/PPC blend with 30 wt% of PPC is much better defined and shifts to a much lower temperature peaked at 110.2 °C, as also shown in Figure 3, likely due to the increased mobility of PLA component with the inclusion of soft PPC. Based on the melting, the crystallinity of the neat PLA is 7.31%, while it increases to 43.64% for the PLA component for its blend with 30 wt% of PPC, due to the increased mobility of PLA component with the inclusion of soft PPC.

It is surprised that for the PLA/PPC blend with 1 or 2 wt% of TBT, no PLA cold crystallization is observed, as shown in Figure 3; this indicates that the PLA component in these blends can fully crystallize during the cooling process (Figure 5) with a crystallinity of 42–43% (Table 4). For the blend with 3 wt% of TBT, a large cold crystallization peak shows up again at 107.5 °C, similar to the PLA/PPC blend, since the crystallinity of this blend during the cooling process is very limited (3%) and hence significant cold crystallization occurs during the subsequent heating process. As mentioned earlier, 3 wt% of TBT might induce too much PLA-PPC copolymer, which makes the PLA rich-phase much smaller and hence weakens the crystalline ability of PLA component. The PLA component in PLA/PPC blend and in all blends with TBT have much higher crystallinity ranging from 42% to 44%, which is so much higher than the neat PLA (7.3%), as listed in Table 5.

Figure 5 shows that crystallization dynamics and the crystallinity of the blend with 2 wt% of TDI is very similar to the blend with 3 wt% of TBT, but their melting behavior is very different as shown in Figure 3. The blend with 2 wt% of TDI shows a similar cold crystallization as the neat PLA sample during the heating (Figure 3), but at a slightly lower temperature, indicating that the crystalline ability of the PLA component is constrained during the previous cooling process, which is different from the blends with 1–2% of TBT The overall crystallinity of PLA component in this blend with 2 wt% TDI is significantly lower (33.6%) than any PLA/PPC blend with or without TBT (42–44%), which, in fact, can be interpreted with phase structure model shown in Figure 4. On one hand, the fact that the copolymer consists of 70% of PPC (see our previous calculation with Fox equation and the *Tg* of copolymer interphase) suggests that the PLA in the copolymer is very likely unable to crystallize. On the other hand, the PLA-PPC copolymer can act as the interphase between the PLA and PPC phases in the blend, which can improve their compatibility of the blend, and hence more and smaller PPC dispersed domains can be formed in the PLA matrix, which can also contribute to a weaker crystalline ability and lower crystallinity of PLA matrix.

### 3.3. Effects of TBT and TDI on the Lamellar Packing Structure of the PLA/PPC Blends

**Figure 6:**
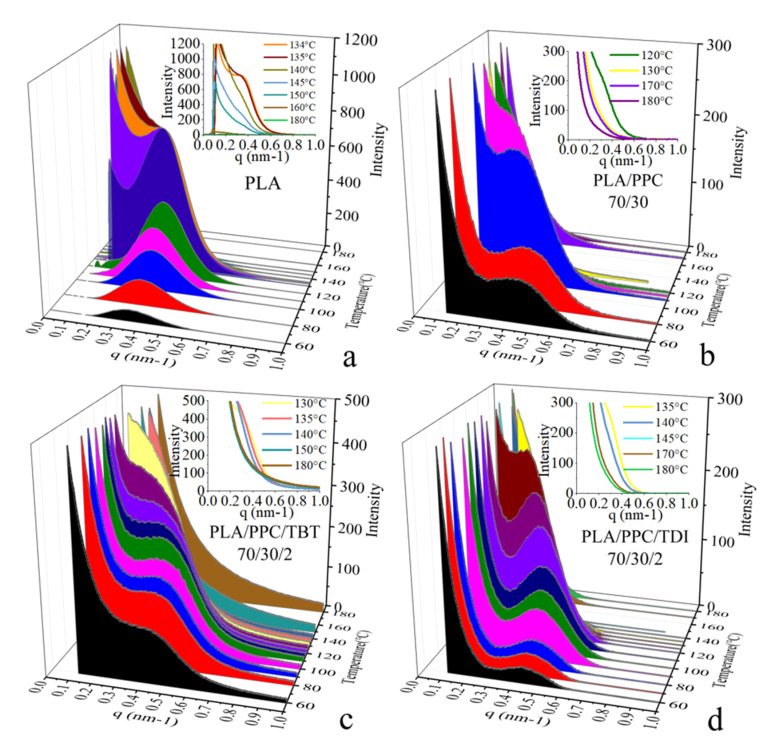
Selected in situ SAXS profiles during a cooling process at 4 °C/min of PLA (**a**), PLA70/PPC30 blend (**b**), PLA70/PPC30/TBT2 blend (**c**) and PLA70/PPC30/TDI2 blend (**d**).

Time-resolved crystallization is also studied with situ synchrotron SAXS/thermal stage in this work. Figure 6 shows the time-resolved SAXS profiles during a cooling process at a rate 4 °C/min of PLA, PLA/PPC blend, and the PLA/PPC blends with 2wt% of TBT and TDI, respectively. It is seen from the inset figure in Figure 6a that during the cooling process of the neat PLA from 180 °C to 140 °C, the SAXS scattering intensity increases with lowering the temperature, and a clear scattering shoulder around q = 0.4 nm^–1^ appears at 135 °C, indicating organized lamellar stacks are formed at this temperature. A well-defined scattering peak is observed when the temperature reaches to 130 °C (Figure 6a), indicating the formation of very ordered PLA lamellar stacks. With further lowering the temperature to 120 °C and below, the scattering intensity drops significantly while the peak width becomes broader; this is likely due to smaller lamellar stacks are formed at low temperature range, which makes the correlation of overall lamellar stacks less organized, contributing to the lower scattering intensity.

It is also observed from the in situ SAXS experiment that the crystallization rate of the PLA/PPC blend is slower than the neat PLA. It shows a scattering shoulder at a much lower temperature (114 °C, Figure 6b) than the neat PLA, indicating the hindered crystallization process due to the inclusion of a fraction of PPC in the PLA phase in this blend. On the other hand, the scattering peak observed at and below 110 °C for the blend is less well defined than the neat PLA. In fact, as shown in Figure 6b that the blend also shows a strong scattering near beam stop region, which can be attributed to the scattering of dispersed PPC phase due to the incompatibility of the blend. The SAXS profiles of the PLA/PPC blend with 2 wt% TBT during the cooling process are shown in Figure 6c and a scattering shoulder is observed at 130 °C, which is significantly higher than the blend without TBT, which is consistent with the DSC cooling study (Figure 5). The crystallization at a significantly higher temperature is likely due to the nucleation role of the improved interphase of PLA and PPC phases. Compared with the PLA/PPC blend without TBT, the SAXS scattering of the blend with 2 wt% TBT is much less well-defined during the whole cooling process and only a scattering shoulder is observed. The absence of well-defined scattering peak for the blend with 2 wt% TBT indicates that the PLA lamellar packing in this blend is significantly less ordered than the PLA/PPC blend, presumably resulted from the improved compatibility of PLA with much smaller dispersed PPC domains in the PLA continuous phase. The PLA/PPC blend with 2 wt% of TDI, however, shows similar well-defined scattering peak as the PLA/PPC blend (Figure 6b,d). It is seen from Figure 6d that a SAXS scattering shoulder is observed around 130 °C during the cooling process, which is similar to the blend with 2% of TBT.

**Figure 7:**
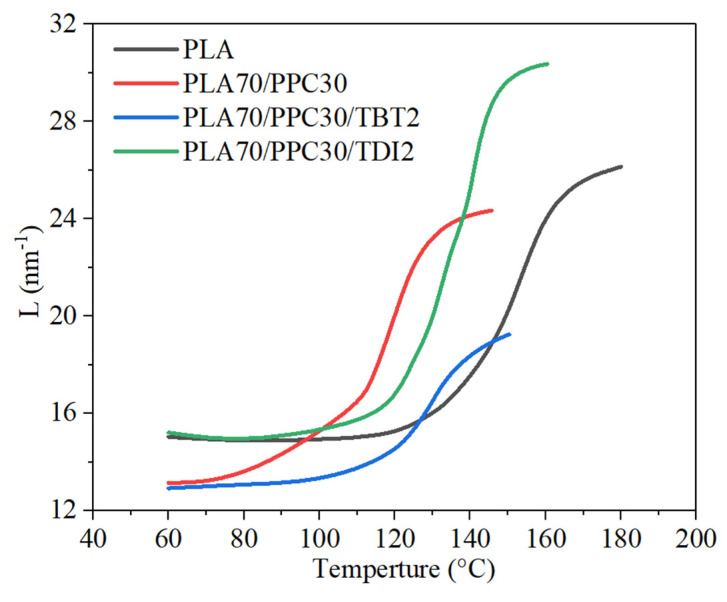
Long period (evaluated with Lorentz-corrected SAXS profiles) vs. cooling temperature during an in-situ DSC and SAXS cooling process at 4 °C/min of the neat PLA, PLA70/PPC30 blend, PLA70/PPC30/TBT2 blend and PLA70/PPC30/TDI2 blend.

The in-situ study of the four selected samples with synchrotron SAXS/thermal stage was carried out to investigate the formation of PLA crystal lamellar stacks, which is actually a further study of crystallization dynamics during a cooling process. The scattering related to the formation of lamellar stacks appears at higher temperature indicates the sample crystallizing earlier during the cooling process. Even though the cooling rate for the SAXS study is slower (−4 °C/min) than the DSC study (−10 °C/min) due to more time is needed to acquire the scattering data for each frame, the crystallization dynamics revealed by SAXS is consistent with the non-isothermal crystallization studied with DSC shown in Figure 5. It can be seen from Figure 7 that the initial formation of lamellar stacks for the neat PLA occurs at a significantly higher temperature than the three blend samples. Figure 7 also shows that the lamellar stacks of the PLA in the blends with 2% TBT and 2% TDI, respectively, are formed at higher temperature than that of the PLA/PPC blend (~130 °C), likely due to improved compatibility between PLA and PPC phases due to added TBT or TDI, leading to earlier nucleation and crystallization likely induced by the improved interphase. 

On the other hand, the long period of PLA alternating lamellar stacks is also quite different for these samples. It is seen from Figure 7 that the initial long period of the PLA/PPC blend at high temperature end is only slightly smaller than the neat PLA (~26 nm), but the final long period of the blend at 60 °C is much smaller than the neat PLA (13.1 vs. 15.0 nm), due to the effects of some compatibility of PLA and PPC and the PPC dispersed domains. The blends with 2% of TBT or TDI have very different initial long period at high temperature end (Figure 7) than the PLA/PPC blend. The blend with 2% of TBT shows a significantly smaller initial long period at ~140 °C (~20 nm) than that of the neat PLA (~25 nm); the final long period of this blend at 60 °C is also significantly smaller than the neat PLA but about the same as the PLA/PPC blend. The significantly smaller initial long period of the blend with 2% of TBT than the PLA/PPC blend is presumably caused by more and smaller dispersed PPC domains due to improved compatibility, which leads to the PPC component being excluded from the PLA lamellar stacks and smaller lamellar stacks are formed. However, the initial PLA long period (~30.5 nm) during the cooling process for the blend with 2% of TDI is much larger than all other two blends, as well as the neat PLA, as shown in Figure 7, and the final long period of the blend is about the same as the neat PLA, but significantly larger than the other two blends. It is not clear why the initial PLA lamellar stacks in the blend with 2% TDI have a much larger long period; it could mean that the formation of initial lamellar stacks is a little more difficult than the other samples, so that less lamellae are formed initially which leads to a larger long period.

### 3.4. Effects of TBT and TDI on Mechanical Property of the PLA/PPC Blends

**Figure 8:**
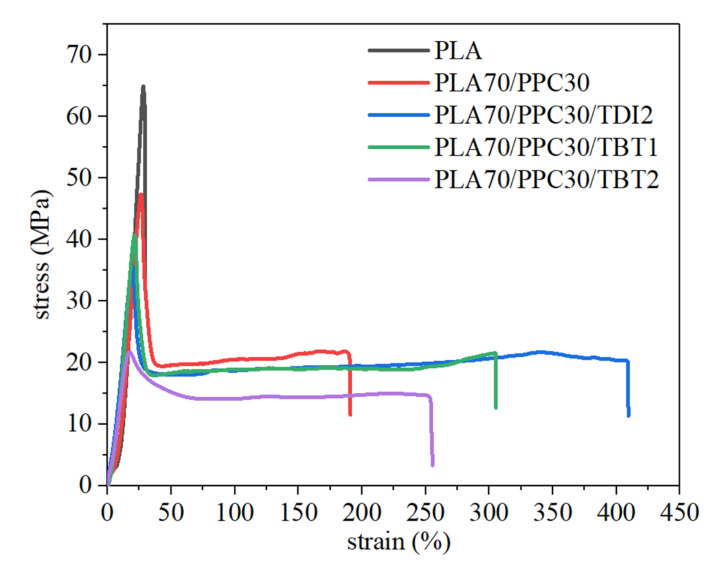
Tensile curves of PLA and PPC, its blend with PPC and the blends with 1–2% of TBT or 2% of TDI.

The mechanical properties of the neat PLA, PLA/PPC blend and the blends with TBT and TDI are also studied in the present work (Figure 8) and interpreted with characterization above. The blend with 3% of TBT shows severe degradation as discussed in earlier section and is dropped for the mechanical test. It can be seen from this figure and Table 6 that the elongation at break of the PLA/PPC blend is dramatically larger than the neat PLA (191% vs. 30%), due to the effect of soft PPC component. The yield strength and fracture strength of the blend drop significantly, compared with neat PLA. The blend with 1% of TBT shows more than 60% larger elongation at break than the PLA/PPC blend, though the mechanical strength of the blend with TBT is slightly lower than the PLA/PPC blend (41 vs. 47 MPa). The yield strength and the fracture strength of the blend with 1% TBT is also lower than the PLA/PPC blend, as shown in Table 6. With further increase in TBT content to 2%, both the elongation at break of the blend and its mechanical strength decreases significantly, as shown in Figure 8, indicating that the material degradation can make larger impact to this blend than the improved compatibility.

Table 6 also shows that compared with neat PLA, the stored energy of the PLA/PPC dramatically increases (720 vs. 3954). The blend with 2 wt%TDI shows the largest stored energy and it is two times larger than the blend without TBT and TDI. The blend with 1 wt% of TBT also shows a significantly increase in stored energy (about 1.5 times larger than the blend without TBT and TDI. The stored energy decreases quickly with more TBT added to the blend due to molecular degradation. The blend with 2% of TDI exhibits the best mechanical property with the elongation at break being 114% larger than the PLA/PPC blend. The yield strength, yield strain and fracture strength of the blend with 2% of TDI is only slightly lower than the blend with 1% of TBT. Therefore, the 70%PLA/30%PPC blend with addition of 2% TDI and the 70%PLA/30% PPC blend with addition of 1% TBT show the best overall mechanical properties. Compared with 70%PLA/30%PPC blend, the elongation at break of the above two blends can be dramatically larger with a slightly lower mechanical strength.

## 4. Conclusions

In this work, PLA-PPC block copolymers are produced in incompatible PLA/PPC blend by initiating transesterification with added 1–2% of TBT or by chain extension with addition of 2% of TDI. The improved compatibility of the blend, the copolymer effect on its crystallization dynamics, PLA lamellar packing structure, thermal and mechanical properties are investigated, including an interpretation of its structure-property relationship. The key conclusions can be summarized as follows.

1. A small fraction of PLA -PPC block copolymer is likely formed in the PLA/PPC blend with addition of 1–2% of TBT due to transesterification, and the block copolymer can act as the compatibilizer at the interface between PLA and PPC phases to improve the compatibility of the blends and hence enhance its mechanical properties.

2. The effect of added TDI on the PLA/PPC blend is quite different. The addition of TDI triggers the chain extension reaction between PLA and PPC and a significant amount of PLA-PPC block copolymer with PPC as a major content (about 70 wt%) is presumably formed in the PLA/PPC blend. The significant amount of the PLA-PPC block copolymer formed in PLA/PPC blend with 2% of TDI is likely to have a complicated grafting structure; the copolymer forms a miscible phase between PLA and PPC phases with its own Tg. This 3rd phase between PLA and PPC phase plays an important role for much better overall mechanical properties of this blend than the 70%PLA/PPC blend.

3. Material degradation can occur for 70%PLA/PPC blend with addition of TBT due to the effect of transesterification; however, with addition of 1 wt% of TBT, the blend shows limited degradation and the overall mechanical property improved very significantly compared with the PLA/PPC blend without TBT, especially for the elongation ratio at break. However, no degradation is observed for the blend with addition of TDI, moreover, there is a significant increase in molecular weight for the 70%PLA/PPC blend with 2% of TDI due to chain extension reaction. As the result, the blend with 2% of TDI has the best overall mechanical properties. 

4. The crystalline ability of neat PLA is very weak during the cooling process. The addition of PPC increases mobility of PLA component in the blend, and hence improves the crystalline ability of PLA component. The crystalline ability of PLA/PPC blends with 1–2%TBT increases sharply during the cooling process; as the result, the cold crystallization, seen during a subsequent heating process for the PLA/PPC blend, is no longer present for these blends. The crystallization of the PLA/PPC blend with 2% of TDI during a cooling process does not change that much with added TDI, but its crystallinity is significantly lower than the blend without TDI, as the PLA blocks in the copolymer with PPC being the major content is hardly to crystallize. 

5. In situ synchrotron SAXS study shows that the PLA/PPC blend with 2 wt% of TDI can have a well-defined scattering peak as the PLA/PPC blend, suggesting that the produced copolymer in the interphase does not have that much effect on PLA lamellar packing in this blend, though the PLA crystallinity decreases a lot based on the thermal analysis. However, the PLA lamellar packing in the blend with 2% of TBT becomes significantly less ordered than the PLA/PPC blend, possibly caused by much smaller PPC domains formed in this blend due to the better compatibility contributed by both the copolymer and some degree of material degradation. 

6. The 70%PLA/30%PPC blend with 2% of TDI or 1% TBT can have much better mechanical properties than 70%PLA/30%PPC blend; the elongation at break of the above two blends can be dramatically larger with a slightly lower mechanical strength. The 70%PLA/30%PPC blend with 2% of TDI exhibits the best mechanical property with the elongation at break being 114% larger than the PLA/PPC blend. The next is the 70%PLA/30%PPC blend with 1% TBT, which shows about 60% larger elongation at break than the PLA/PPC blend. 

## Figures and Tables

**Table 1:** The compositions of the PLA/PPC/TDI and PLA/PPC/TBT blends.

Sample Designation	PLA (wt%)	PPC (wt%)	Compatibilizer	
			TDI (wt%)	TBT (wt%)
PLA	100	0	0	0
PPC	0	100	0	0
PLA70/PPC30	70	30	0	0
PLA70/PPC30/TBT1	70	30	0	1
PLA70/PPC30/TBT2	70	30	0	2
PLA70/PPC30/TBT3	70	30	0	3
PLA70/PPC30/TDI2	70	30	2	0

**Table 2:** Melt index values of PLA70/PPC30 blends with TBT or TDI.

Sample	Melt Flow Rate (g/10 min)
PLA70/PPC30	15.6 ± 0.8
PLA70/PPC30/TBT1	20.5 ± 1.1
PLA70/PPC30/TBT2	68.4 ± 2.7
PLA70/PPC30/TBT3	302.1 ± 12.3
PLA70/PPC30/TDI2	4.5 ± 0.2

**Table 3:** Glass transition temperature and change in heat capacity for Tg transition.

Sample	Miscible Blend		PPC-Rich Phase		PLA-Rich Phase		PLA-PPC Copolymer Phase	
	Tg (°C)	ΔC_p_ (J/g·°C)	Tg (°C)	ΔC_p_ (J/g·°C)	Tg (°C)	ΔC_p_ (J/g·°C)	Tg (°C)	ΔC_p_ (J/g·°C)
PLA					61.0 ± 0.3	+0.61 ± 0.04		
PPC			30.5 ± 0.2	+0.93 ± 0.05				
PLA70/PPC30			37.5 ± 0.4	+0.28 ± 0.02	59.8 ± 0.2	+0.59 ± 0.01		
PLA70/PPC30/TBT1			33.6 ± 0.2	+0.34 ± 0.02	57.9 ± 0.1	+0.24 ± 0.03		
PLA70/PPC30TBT2			32.7 ± 0.1	+0.33 ± 0.01				
PLA70/PPC30/TBT3	51.8 ± 0.2	+0.53 ± 0.05						
PLA70/PPC30/TDI2			31.2 ± 0.1	+0.29 ± 0.02	62.2 ± 0.4	+0.43 ± 0.01	39.4 ± 0.2	+0.36 ± 0.01

**Table 4:** Crystallization temperature and crystallinity of PLA in the PLA/PPC blends during a cooling process at 10 °C/min.

Sample Designation	PLA/PPC/TBT or TDI (wt%)	T_c_ (°C)	ΔH_c_ (J/g)	X_c_ (%)
PLA	100/0/0	100.1 ± 0.2	0.86 ± 0.05	0.92 ± 0.03
PLA70/PPC30	70/30/0	92.9 ± 0.1	2.74 ± 0.12	4.17 ± 0.21
PLA70/PPC30/TBT1	70/30/1	106.5 ± 0.3	27.73 ± 0.23	42.31 ± 0.37
PLA70/PPC30/TBT2	70/30/2	107.0 ± 0.2	28.40 ± 0.17	43.34 ± 0.25
PLA70/PPC30/TBT3	70/30/3	90.9 ± 0.1	1.97 ± 0.11	3.00 ± 0.12
PLA70/PPC30/TDI2	70/30/2	98.5 ± 0.1	15.17 ± 0.21	23.16 ± 0.24

**Table 5:** Cold crystallization temperature, cold crystallization enthalpy and melt enthalpy of the PLA/PPC blends during a reheating process at 10 °C/min.

Sample	T_cc_ (°C)	ΔH_cc_ (J/g)	T_m_ (°C)	ΔH_m_ (J/g)	X_c_ (%)
PLA	138.8 ± 0.2	6.02 ± 0.22	167.3 ± 0.2	6.9 ± 0.2	7.31 ± 0.21
PPC		-	-	-	-
PLA70/PPC30	110.2 ± 0.2	24.46 ± 0.37	169.2 ± 0.1	28.6 ± 0.3	43.64 ± 0.32
PLA70/PPC30/TBT1	-	-	167.8 ± 0.1	28.1 ± 0.2	42.88 ± 0.21
PLA70/PPC30/TBT2	-	-	163.0 ± 0.3	28.7 ± 0.1	43.8 ± 0.11
PLA70/PPC30/TBT3	107.5 ± 0.3	25.21 ± 0.42	165.1 ± 0.1	27.6 ± 0.2	42.04 ± 0.21
PLA70/PPC30/TDI2	126.3 ± 0.1	6.23 ± 0.16	166.7 ± 0.2	22.0 ± 0.1	33.57 ± 0.11

**Table 6:** Mechanical property data of PLA, its blend with PPC and the blends with 1–2% of TBT or 2% of TDI.

Sample	Yield Strength (MPa)	Yield Strain (%)	Fracture Strength (MPa)	Elongation at Break (%)	Stored Energy up to Breaking
PLA	65.19 ± 1.53	28.12 ± 1.47	60.81 ± 2.85	29.58 ± 4.25	720.32 ± 22.62
PLA70/PPC30	47.39 ± 2.24	26.45 ± 1.13	18.56 ± 2.02	190.84 ± 19.73	3954.42 ± 171.38
PLA70/PPC30/TBT1	40.73 ± 1.48	21.24 ± 1.67	21.52 ± 1.37	305.20 ± 21.35	5878.96 ± 316.26
PLA70/PPC30/TBT2	21.72 ± 1.47	17.49 ± 1.88	14.23 ± 0.48	255.42 ± 16.48	3725 ± 193.74
PLA70/PPC30/TDI2	35.62 ± 1.34	19.58 ± 0.21	19.98 ± 0.44	409.17 ± 27.27	8066.71 ± 449.72

## Data Availability

The data presented in this study are available on request from the corresponding author.
